# An explorable model of an adverse outcome pathway of cytokine release syndrome related to the administration of immunomodulatory biotherapeutics and cellular therapies

**DOI:** 10.3389/fimmu.2025.1601670

**Published:** 2025-08-08

**Authors:** Alexander Mazein, Oxana Lopata, Kristin Reiche, Katherina Sewald, Miriam Alb, Christina Sakellariou, Patricia Gogesch, Hannah Morgan, Vanessa Neuhaus, Nhu-Nguyen Pham, Charline Sommer, Ethan Perkins, Birgit Fogal, Muhammad Shoaib, Reinhard Schneider, Venkata Satagopam, Marek Ostaszewski

**Affiliations:** ^1^ Luxembourg Centre for Systems Biomedicine, University of Luxembourg, Belvaux, Luxembourg; ^2^ Department of Diagnostics, Fraunhofer Institute for Cell Therapy and Immunology IZI, Leipzig, Germany; ^3^ Institute for Clinical Immunology, University Hospital of Leipzig, Leipzig, Germany; ^4^ Center for Scalable Data Analytics and Artificial Intelligence (ScaDS.AI), Dresden, Leipzig, Germany; ^5^ Department of Preclinical Pharmacology & Infection and Immunology, Fraunhofer Institute for Toxicology and Experimental Medicine, Hannover, Germany; ^6^ Member of German Center for Lung Research, Biomedical Research in Endstage and Obstructive Lung Disease Hannover (BREATH), Member of Fraunhofer Cluster of Excellence Immune-Mediated Diseases (Fraunhofer CIMD), Hannover, Germany; ^7^ Universitätsklinikum Würzburg, Medizinische Klinik und Poliklinik II, Lehrstuhl für Zelluläre Immuntherapie, Würzburg, Germany; ^8^ Department of Immunotechnology, Lund University, Lund, Sweden; ^9^ Division of Immunology, Paul-Ehrlich-Institut, Langen, Germany; ^10^ Preclinical Safety, Novartis Biomedical Research, Basel, Switzerland; ^11^ Preclinical Pharmacology and In-Vitro Toxicology, Fraunhofer Institute for Toxicology and Experimental Medicine (Fraunhofer ITEM), Hannover, Germany; ^12^ Biomedical Research in Endstage and Obstructive Lung Disease Hannover (BREATH), Member of the German Center for Lung Research (DZL) and Fraunhofer Cluster of Excellence Immune-Mediated Diseases (Fraunhofer CIMD), Hannover, Germany; ^13^ Department of Vaccines and Infection Models, Fraunhofer Institute for Cell Therapy and Immunology IZI, Leipzig, Germany; ^14^ Immunology & Immunotoxicology, Labcorp Drug Development, Harrogate, United Kingdom; ^15^ Institute of Cancer Therapeutics, Faculty of Life Sciences, School of Pharmacy and Medical Sciences, University of Bradford, Bradford, United Kingdom; ^16^ Nonclinical Drug Safety, Boehringer Ingelheim Pharmaceutical, Inc., Ridgefield, CT, United States; ^17^ European Life-sciences Infrastructure for Biological Information (ELIXIR) Luxembourg, Belvaux, Luxembourg

**Keywords:** systems biology, systems toxicology, immunomodulatory therapies, adverse outcome pathway (AOP), cytokine release syndrome (CRS), CAR T cells

## Abstract

**Introduction:**

Cytokine release syndrome (CRS) is a potentially severe systemic inflammatory condition triggered by various immunomodulatory therapies, making understanding its pathogenesis critical for improving patient outcomes.

**Results/Methods:**

By combining immunotoxicology and systems biology approaches, we offer a novel and integrative conceptual model of CRS as an adverse outcome (AO), induced by five different immunomodulatory biotherapies: 1) chimeric antigen receptor (CAR) T cells, 2) checkpoint inhibitors, 3) T cell engaging bispecific modalities, 4) monoclonal antibodies targeting and activating T cell receptors, and 5) FcγR activating monoclonal antibodies. This model uniquely integrates multiple CRS-inducing therapies into a unified framework, offering a comprehensive mechanistic representation of CRS pathophysiology. For that, we built an adverse outcome pathway (AOP) CRS network for these therapies and then developed a systems biology map of molecular mechanisms relevant to the AOP network. The map of mechanisms is made available via a dedicated online platform for exploration and data visualisation. It includes 24 cell types, 425 entities and 430 interactions.

**Discussion:**

Beyond a static representation, the CRS Map serves as a dynamic tool for clinical and research applications, allowing researchers and clinicians to explore CRS progression in detail, identify biomarkers, and discover potential therapeutic targets. The map demonstrates stages of CRS progression and shows molecules that can be measured in relevant immunotoxicological assays, as well as potential drug targets for therapeutic intervention of CRS.

## 1 Introduction

The nonclinical safety assessment of immunomodulatory biotherapies still faces many challenges, primarily due to the complex interactions these drugs have with the components and functions of the human immune system. Strategies to define immune-related adverse outcome pathways (irAOPs) guide the way for the seamless incorporation of this complexity into novel testing strategies. Adverse Outcome Pathways (AOPs) are a conceptual framework to support scientists with diverse expertise to systematically understand the biological mechanisms underlying adverse effects following exposure to various stressors (1–4).

The systematic irAOP approach is particularly beneficial in cases where undesired drug-induced immune pathways involve complex crosstalk, such as in cytokine release syndrome (CRS). CRS, initially termed in the 1990s during the clinical development of the anti-CD3 monoclonal antibody OKT3 (5), is a life-threatening systemic inflammatory disorder. It can occur as a response to various stressors such as viral or bacterial infections (e.g. SARS-CoV-2) (6), or following treatment with immunomodulatory biotherapies (4, 7). CRS is a significant adverse outcome of various immunomodulatory therapies, including CAR T cells, bispecific T cell engagers (TCEs), checkpoint inhibitors (CPIs) (8), and monoclonal antibodies activating immune cells through direct activation of target receptors on T cells or Fcγ receptors (9). On the molecular level, the initial trigger for CRS induced by these biotherapies and cellular therapies involves the activation of adaptive immune cells, most often CD4+ and CD8+ T cells, which leads to a release of proinflammatory mediators by these cells such as interferon gamma (IFN-γ), interleukin (IL-2), and tumour necrosis factor alpha (TNF-α) (10, 11). This initial release of cytokines leads to further recruitment and activation of bystander (innate) immune cells such as monocytes and macrophages which can then also release additional cytokines such as IL-6, one of the key mediators of CRS (12). In turn, various cytokines, including IL-6, can activate endothelial cells. All these mechanisms can result in systemic inflammation, vascular leakage, multi-organ dysfunction and failure (10, 11).

Here we propose a human-readable digital model of immunological mechanisms leading to CRS as an adverse outcome of selected therapies. This digital model was developed based on our novel immunotoxicology approach where the AOP concept (2) is implemented using detailed, diagrammatic pathway-level descriptions of molecular mechanisms (disease maps) (13, 14). Given the complexity of CRS and the importance of the therapies it is associated with, it is important to assemble information about molecular and cellular interactions involved in CRS, in a way that allows intuitive exploration by domain experts and effective computational analysis. Our model offers a systematic and standardised review of pertinent adverse outcome mechanisms, facilitating their deeper understanding and potentially guiding more effective interventions and thus potential CRS-treatment approaches. By its visual layer, it facilitates exploration and review by domain experts, and by its computational capabilities, it enables data integration and bioinformatic analyses.

In this paper, we show the integrated AOP network, describe the new online CRS Map resource while providing contextual information about relevant biology, specify how the resource was built, and demonstrate a use case with its application for data visualisation.

## 2 Results

**Figure 1:**
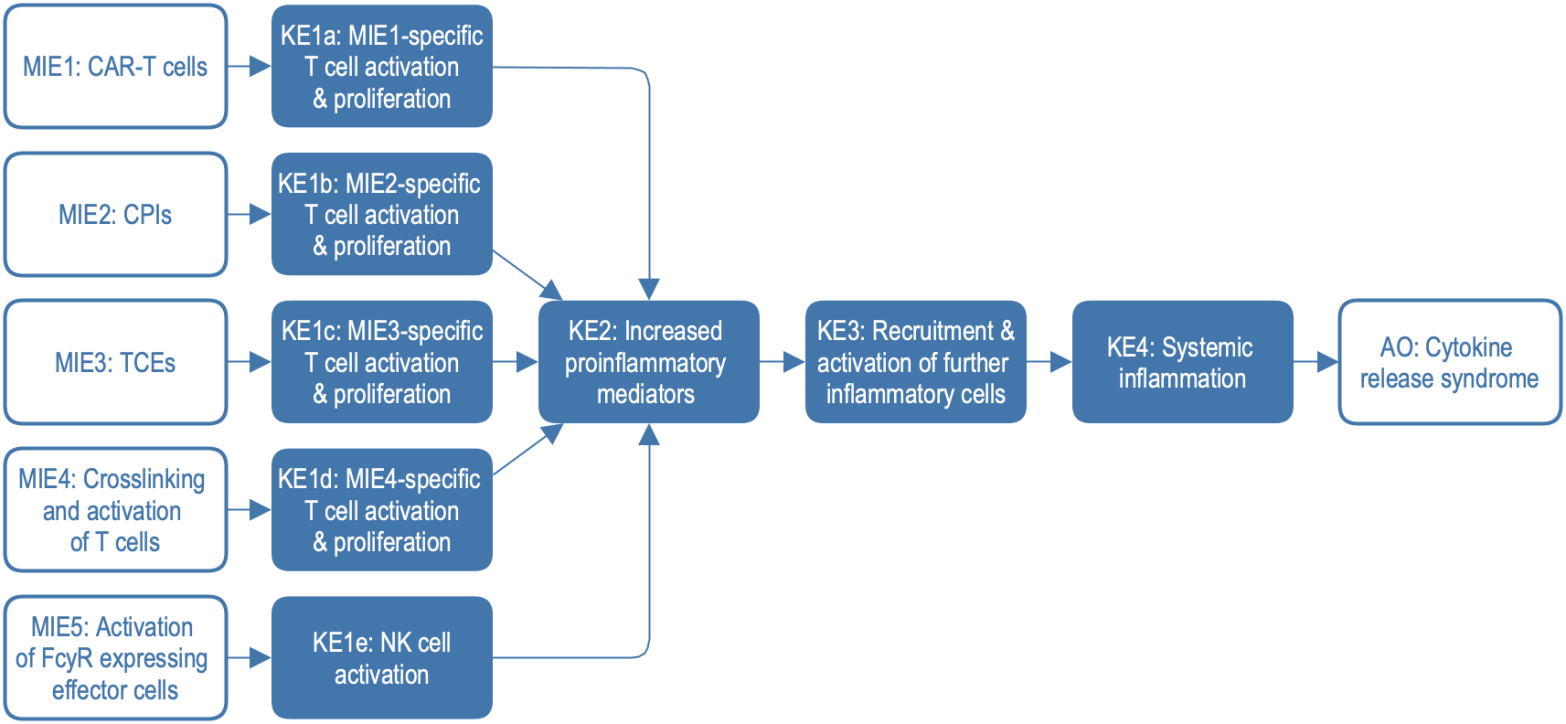
Adverse outcome pathway (AOP) network with five different molecular initiating events. Clinical phenotypes of CRS include mild to severe symptoms including e.g. fever, hypotension, hypoxia, organ failure, and coagulopathy. AO, adverse outcome; CPIs, checkpoint inhibitors; KE, key event; MIE, molecular initiating event; NK, natural killer cells; TCEs, T-cell engagers; FcγR, Fc gamma receptor.

The construction of the irAOP map was driven by an intensive collaborative effort involving both domain experts and computational biologists during a four-day hybrid workshop (study-a-thon) organised under the imSAVAR project (https://imsavar.eu). Through a structured schedule and interdisciplinary environment, the team developed a standardised and annotated representation of cellular and molecular mechanisms implicated in cytokine release syndrome. This iterative exchange enabled the systematic identification and annotation of key entities organised in the connectivity summary table (**Supplementary Table S1**) necessary to develop a model of CRS (**Figure 1**).

To construct the explorable model of CRS, we harmonised and combined three irAOPs for CRS mediated by CAR T cells, bi-specific T cell engagers, and CPIs. Additionally, two further mechanisms associated with CRS were integrated, namely crosslinking and activation of T cells and the activation of FcγR-expressing effector cells. Based on this network of AOPs (**Figure 1**), we constructed a systems biology map by connecting the description of key events (KEs) and key event relationships (KERs) to the representation of the underlying biological mechanisms on molecular and physiological levels. We introduced standard systems biology formats, stable identifiers, and knowledge provenance, enabling versioning, reproducibility, comparability, and compliance with FAIR (Findable, Accessible, Interoperable, and Reusable) principles (https://www.go-fair.org/fair-principles) (15). In this process, we identified and addressed knowledge gaps at the interfaces between different AOPs. Finally, we demonstrated the application of the CRS Map for data visualisation and interpretation using datasets from recent publications.

### 2.1 Harmonisation of CRS AOPs

The irAOP network of CRS-related events was composed of five parts, as illustrated in **Figure 1**. Three of them were irAOPs of CRS mediated by CAR T cells, CPIs, and bi-specific T cell engagers, which were selected for harmonisation. Further, two mechanisms associated with CRS were included: crosslinking and activation of T cells and the activation of FcγR-expressing effector cells. This resulted in an irAOP network with five different molecular initiating events (MIE1-5; for short descriptions and examples, also see **Table 1**) and five corresponding first key events (KE1a-e), which then merged into shared KE2–4 and CRS as an adverse outcome.

**Table 1:** Molecular initiating events of five different therapies with their description and examples of the corresponding drugs.

Therapy	Event	Description	Examples
CAR-T cells	MIE1	Binding of CAR to antigen	Abecma (idecabtagene vicleucel; target: BCMA), Kymriah (tisagenlecleucel; target: CD19), Yescarta (axicabtagene ciloleucel; target: CD19); Tecartus (brexucabtagene autoleucel; target: CD19), Carvykti (ciltacabtagene autoleucel; target: BCMA), Breyanzi (lisocabtagene maraleucel; target: CD19)
Checkpoint inhibitors (CPIs)	MIE2	Binding of CPI to target T cell and formation of immune synapse	Nivolumab (PD-1), Pembrolizumab (PD-1), Avelumab (PDL-1), Ipilimumab (CTLA-4),
T-cell engagers (TCEs)	MIE3	Binding of TCE to both target T cell and antigen expressing cells, and formation of immune synapse	Blinatumomab (CD19), Teclistamab (BCMA), Mosunetuzumab (CD20), Epcoritamab (CD20), Glofitamab (CD20), Elranatamab (BCMA), Talquetamab (GPRC5D)
Crosslinking and activation of T cells	MIE4	Binding of T cell agonist mAb to T cell	Muromonab (OKT3, target: CD3), TGN1412 (CD28)
Activation of FcγR-expressing effector cells	MIE5	Binding of mAb to antigen-expressing cell, interaction of the Fc part of the mAb with FcγR expressing cells, activation of effector functions by FcγR expressing cells	Rituximab (CD20), Alemtuzumab (CD52)

CAR-T cells, chimeric antigen receptor T cellsl MIE, molecular initiating event; CPIs, checkpoint inhibitors; PD-1, programmed cell death protein 1; PDL-1, programmed death-ligand 1; CTLA-4, cytotoxic T-lymphocyte-associated protein 4; TCEs, T-cell engagers; mAb, monoclonal antibody; FcγR, Fc gamma receptor.

The contents of the CRS AOP network were systematically reviewed in a dedicated workshop, conducted in a hackathon style, called study-a-thon (see Methods). During the event, an intensive interdisciplinary collaboration allowed us to organise, annotate, and harmonise elements and their interactions in the CRS AOP network. In parallel, these interactions were translated into systems biology format and given graphical representation (see Section 2.2 Construction of the CRS irAOP map).

In the following subsections, we describe in more detail the MIEs, KEs, and CRS as the main adverse outcome of the five treatment scenarios. We highlight molecular mechanisms behind transitions between events.

#### 2.1.1 MIE1 & KE1a: CAR T cell treatment

CAR T cells are a type of immunotherapy consisting of CD4+ and CD8+ T cells which are transduced to stably express the CAR of choice, e.g. anti-CD19 (16). CARs are synthetic receptors comprising in most cases a single chain fragment variable (scFv) of a mAb which is specific for a cell surface antigen, e.g. for CD19 (HGNC symbol: CD19) or B-cell maturation antigen BCMA (HGNC symbol: TNFRSF17, synonym: CD269), and costimulatory domains of either CD28 (HGNC symbol: CD28) or 4-1BB (HGNC symbol: TNFRSF9, synonym: CD137) in “second-generation” CARs (17). CAR T cells are used to treat B-cell leukaemia, B cell lymphoma and multiple myeloma (18, 19), with CD19 CAR T cell therapy also showing remarkable results in autoimmune diseases (20). Six different CAR T cell products targeting either CD19 or BCMA have been approved by FDA/EMA (**Table 1**).

The costimulatory domain within the CAR activates the T cell upon binding of its CAR molecules to the respective antigen. The costimulatory domains of the CAR will recruit molecules within the T cells like lymphocyte-specific protein tyrosine kinase (HGNC symbol: LCK) which phosphorylates downstream signalling molecules like the zeta chain of T cell receptor associated protein kinase 70 (HGNC symbol: ZAP70). In conventional T cells this cascade is initiated by recognition of the appropriate antigen presented by other cells via major histocompatibility complex (MHC; or HLA, human leukocyte antigen) class I molecules in CD8+ T cells or MHC class II molecules in CD4+ T cells (“signal 1”) and binding of CD28 and/or 4-1BB/CD137 to its respective counterpart on the antigen-presenting cell (“signal 2”). By combining both elements in the synthetic receptor, CAR T cells can be activated in an MHC-independent manner. This enables them to proliferate and release cytokines such as IL-2 or IFN-γ. CD8+ T cells can also release perforin and granzyme B from granules stored within the cells which will then lyse the antigen-expressing cells.

For CAR-T cell therapy, there are several factors that will determine if CRS is initiated, such as the lymphodepletion regimen employed prior to CAR-T cell infusion, the type of CAR used as well as the initial magnitude of CAR-T cell expansion upon antigen encounter. There is a certain amount of on-target off-tumour effect that further contributes to the onset of CRS in this treatment, as e.g. CD19-specific CAR-T cells not only target the malignant CD19-expressing tumour cells but also normal B cells expressing this antigen (21).

#### 2.1.2 MIE2 & KE1b: checkpoint inhibitors

Multiple co-stimulatory and co-inhibitory receptors (checkpoints) are involved in tight regulation of immune cell activation to control immune responses, with an overall integration of these signals at any given time determining the T cell response (22). Many checkpoint inhibitors (CPIs) are used as immunotherapies for cancer to release natural brakes on the immune response and allow T cells to recognise and kill tumour cells. As described, antigen-dependent T cell activation requires T cell receptor (TCR) activation through antigen presented on MHC by an antigen presentation cell, and through an overall co-stimulatory signal, best described as a secondary signal provided through activation of the CD28 axis (23). The binding of CPI to target T cells (MIE2) inhibits a negative signal that prohibits T cell activation following formation of an immune synapse, activation of the TCR, and integration of all of these given signals. This leads to T cell activation, the release of cytokines (i.e. IL-2 and IFN-γ), and T cell proliferation. Cytotoxic CD8+ T cells can then also release perforin and granzyme to lyse cells.

#### 2.1.3 MIE3 & KE1c: T-cell engagers

T cell engagers (TCE) are a class of engineered multi-specific antibodies designed to recognise a tumour target and redirect T cells to kill the tumour. The formation of an immune synapse through binding of TCE to the TCR and a target cell will result in T cell activation without the requirement of TCR activation through MHC and co-stimulation through additional surface receptors (24). Activated T cell will release cytokines (i.e. IL-2 and IFN-γ) and proliferate. Cytotoxic CD8+ T cells will also release perforin and granzyme to lyse the target cells.

#### 2.1.4 MIE4 & KE1d: crosslinking and activation of T cells

Monoclonal antibodies (mAbs) are used in immunotherapy (25, 26) typically as IgG isotype. Their function is determined by their target specificity and effector function (affinity to different types of FcγR on various immune cells) (27–29). The induction of CRS can be associated with mAbs applied for the treatment of different malignancies, autoimmune diseases, or transplant rejections (30). They include OKT3 (withdrawn since 2010), and TGN1412, a prominent CRS-inducing mAb. They specifically bind to T cell-specific antigens, which are associated with T cell activation, CD3 (compound of the TCR) or CD28 (costimulatory molecule), respectively, and are therefore examples for MIE4 and KE1d (31–33).

As mentioned above, T cell activation requires activation of TCR in the presence of an overall costimulatory signal. In the case of mAb agonists, T cell activation is induced already by only one of the two required signals - TCR-activation or costimulation (34). However, subthreshold priming of the TCR signalling (35), such as cross-linking of the mAb by Fc: FcγR-binding (e.g. for OKT3), or ICOS-ICOSL interaction between T cell and other (immune) cells (e.g. for TGN1412) are necessary for T cell activation, most likely by inducing increased cytokine production (36–38).

#### 2.1.5 MIE5 & KE1e: activation of FcγR expressing effector cells

The ability of mAbs to induce antibody-dependent cellular cytotoxicity (ADCC) via Fc: FcγR-interaction, as described in MIE5 and KE1e, is used to eliminate specific cells during cancer therapy, which can be accompanied by CRS as an adverse outcome. The binding of the mAb to its antigen on the target cell and to the activating FcγRIIIa on the effector cell induces the corresponding FcγR signalling cascade resulting in the secretion of cytokines and cytotoxic effector molecules (36, 39, 40). While this phenomenon is mainly attributed to NK cells, also other immune cells such as neutrophils or eosinophils are potential effector cells inducing antibody-dependent cellular cytotoxicity.

#### 2.1.6 KE2: increased proinflammatory mediators

**Figure 2:**
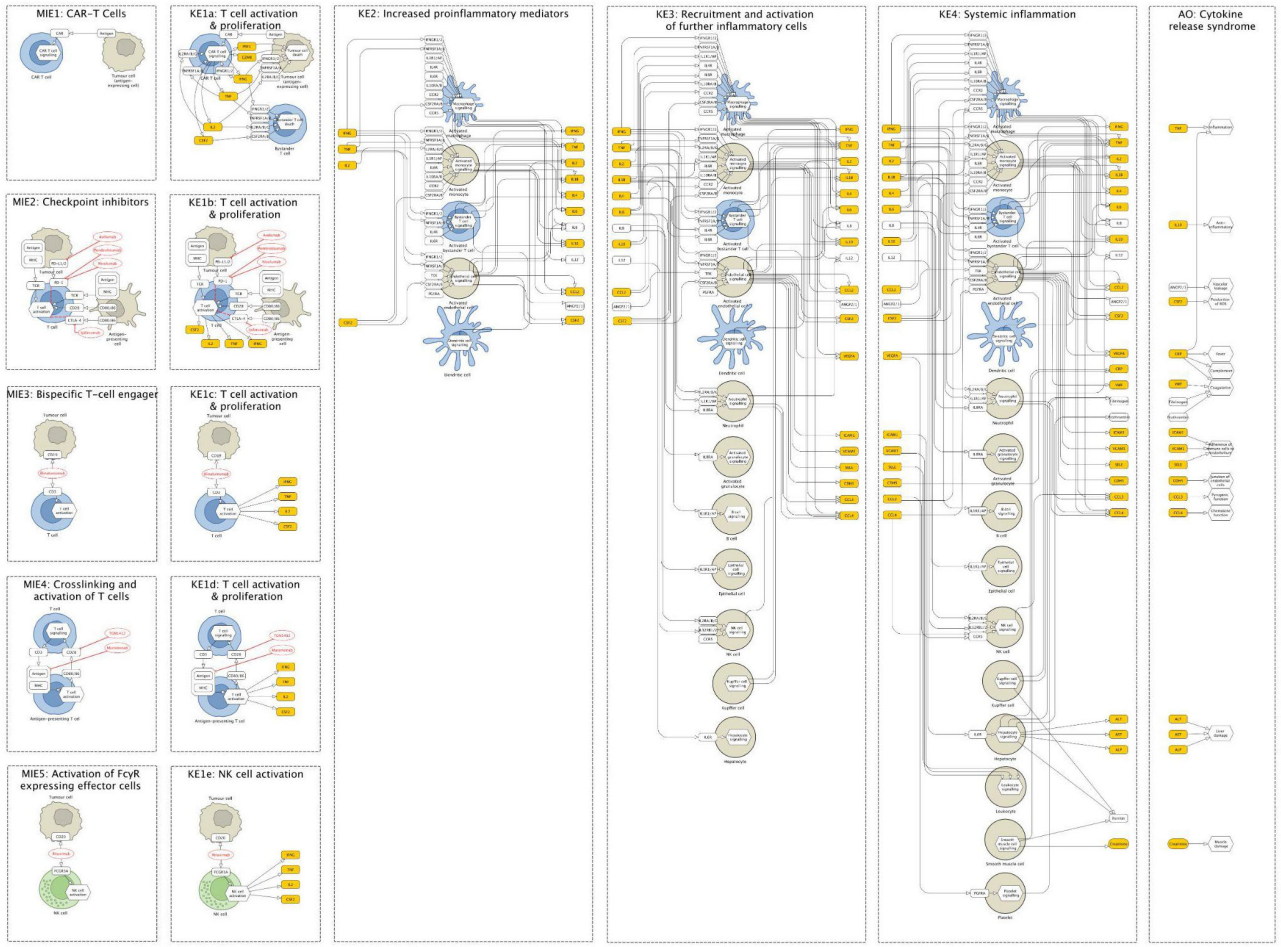
The Cytokine Release Syndrome Map (CRS Map) illustration. The online version of the map is available for browsing and exploration in the MINERVA Platform at https://imsavar.elixir-luxembourg.org/minerva/index.xhtml?id=CRSmap123. Yellow rectangles show specific proteins, and white rectangles show generic proteins (more than one specific protein included in the collection). The map can be downloaded from MINERVA in various formats including SBML and CellDesigner formats (Submaps>Download).

As described above, the MIEs and KE1 result in the release of IL-2, IFN-γ, and TNF-α from T cells. In the case of MIE5, FcγR expressing effector cells are also involved in this step, including NK cells. This initial release of cytokines activates local macrophages, dendritic cells, other bystander immune cells, and non-immune cells like endothelial cells, which then triggers a domino effect, resulting in the release of large amounts of pro-inflammatory cytokines, including IL-6, IL-10, and IFN-γ. This complex interplay is visualised in the flowchart of KE2 (**Figure 2**), which shows the pathways and interactions among various immune cells and cytokines. A hallmark of severe CRS is the pronounced activation of endothelial cells mediated by these pro-inflammatory cytokines and characterised by the release of factors including Angiopoietin-2 (HGNC symbol: Ang-2, synonym: ANGPT2) and von Willebrand factor (HGNC symbol: VWF). These inflammatory mediators, as indicated in the figure, are notably elevated in the blood of patients suffering from CRS.

#### 2.1.7 KE3: recruitment and activation of further inflammatory cells

Immune cells and bystander cells of KE2 produce vast amounts of pro-inflammatory mediators. These mediators, depicted in KE3 (**Figure 2**), activate and recruit more macrophages, T cells, and other immune cells such as granulocytes and NK cells, leading to a positive feedback loop and finally a cytokine storm. The diagram of KE3 shows these interactions, emphasising the signalling pathways that contribute to the escalation of the inflammatory response. In addition, endothelial cells are deeply involved in the CRS pathophysiology, both as an amplifier of the inflammatory response and as target cells, as depicted in KE3 (**Figure 2**). Moreover, the systemic release of these pro-inflammatory mediators leads to the activation of organ-specific cells such as Kupffer cells and hepatocytes, leading to the release of acute phase proteins including e.g. C-reactive protein (CRP) (41) that can contribute to the inflammatory response. These cellular responses culminate in the extensive inflammatory reaction at the point where this normal biological process becomes a pathological reaction.

#### 2.1.8 KE4: systemic inflammation

The release of pro-inflammatory mediators by the multiple cell types described in KE3 leads to a state of pathological, acute inflammation in patients. Prolonged endothelial cell activation leads to cell damage, capillary leakage, which contributes to the release of e.g. creatine and lactate dehydrogenase (LDH). The liver, via the activation of Kupffer cells and hepatocytes, is the major source of acute phase proteins that are the key mediators of systemic inflammation and are responsible for many clinical signs associated with CRS. C-reactive protein can influence the inflammatory process by stimulating myeloid cells, and via the activation of the complement system by binding to components of damaged cells. Fibrinogen is another protein that is strongly increased during systemic inflammation, leading to coagulopathies. Finally, ferritin is a well-described inflammatory mediator and levels observed during systemic inflammation are strongly correlated with the severity of CRS observed in patients.

#### 2.1.9 AO: cytokine release syndrome

Symptoms of CRS can vary significantly in patients following the administration of immunomodulatory therapies. However, fever, hypotension, and hypoxia are the critical symptoms that define the grade of CRS used by clinicians to prescribe treatment (42). High-grade CRS (grades 3-4) requires significant medical interventions due to the involvement of multiple organ systems and life-threatening complications, e.g. cardiac dysfunction, acute respiratory distress syndrome, renal and hepatic failure, and coagulopathies including disseminated intravascular coagulation. In the worst case, CRS can result in death.

### 2.2 Construction of the CRS irAOP map

AOPs are concise and often linear diagrams that consist of several steps. At the same time, their development requires a thorough investigation of relevant biological systems and an intensive literature review. This means that an AOP diagram presents only a part of the information collected for its development, focused on key measurable molecules and key cell types in their relation to major physiological and pathophysiological processes that lead to an adverse outcome. However, especially in the case of complex pathologies, the entirety of collected information and its provenance is necessary. To connect different CRS-related AOPs into a consistent network, we used this extended information and organised it in a structured systematic way to show the underlying biology. This way, we developed a graphical and computational AOP-based model of CRS mechanisms - the irAOP CRS systems biology map (CRS Map). Building a systems biology map based on an AOP or an AOP network brings additional advantages and perspectives:

- connecting the description of key events (KEs) and key event relationships (KERs) to the representation of the underlying biology on molecular and physiological levels;
- applying well-established standard systems biology formats such as Systems Biology Markup Language (SBML) and Systems Biology Graphical Notation (SBGN);
- introducing stable identifiers of the molecules involved, for example, UniProt IDs for proteins and ChEBI IDs for metabolites;
- identifying knowledge gaps as a result of a biological network reconstruction effort;
- enabling versioning, reproducibility and comparability of the CRS Map;
- allowing its further integration with bioinformatic resources, e.g. AOP-Wiki, to make its content reusable and interoperable.

The coverage of the CRS Map was defined by the original AOP network (**Table 1**, **Figure 1**), and included five different MIEs, the effects of which were merged in the description of the next steps and led to the adverse outcome. The coverage and boundaries of the Map were further determined by the AOP connectivity table (see Methods, **Supplementary Table S1**), listing key molecules and cell types, their connectivity and stable identifiers (see an example fragment in **Table 2**).

**Table 2:** An information card for IL-6, an extract from the summary table (**Supplementary Table S1**).

Entity	Value
Name	IL-6
HGNC Name	IL6
UniProt	UniProt:P05231
Category	Soluble
Key events	2, 3, 4
Modulation	Increase of activity
Source cells	Monocytes, macrophages, endothelial cells
Biological consequence	Leads to systemic inflammation, pyrogenic, promotes T effector cell differentiation, promotes CRP and fibrinogen production from hepatocytes
Target cells	Bystander T cells, monocytes, macrophages, hepatocytes
Receptors	IL6R (UniProt:P08887), IL6ST (UniProt:P40189)
References	PMID:27381687, PMID:29387417, PMID:27076371

The ‘entity’ elements in this table correspond to the columns of **Supplementary Table S1** (Name, HGNC Name, etc.). The main purpose of this table is to identify molecules, assign them to the relevant events of the AOP network, determine the source and target cell types, show the corresponding receptors, as well as define the role of molecules in the adverse outcome mechanisms (biological consequence). Databases: HGNC, HUGO Gene Nomenclature Committee, https://www.genenames.org; UniProt, https://www.uniprot.org. IL-6, interleukin 6; UniProt, Universal Protein Resource; IL6R, interleukin 6 receptor; IL6ST, interleukin 6 signal transducer; PMID, PubMed Identifier.

**Figure 3:**
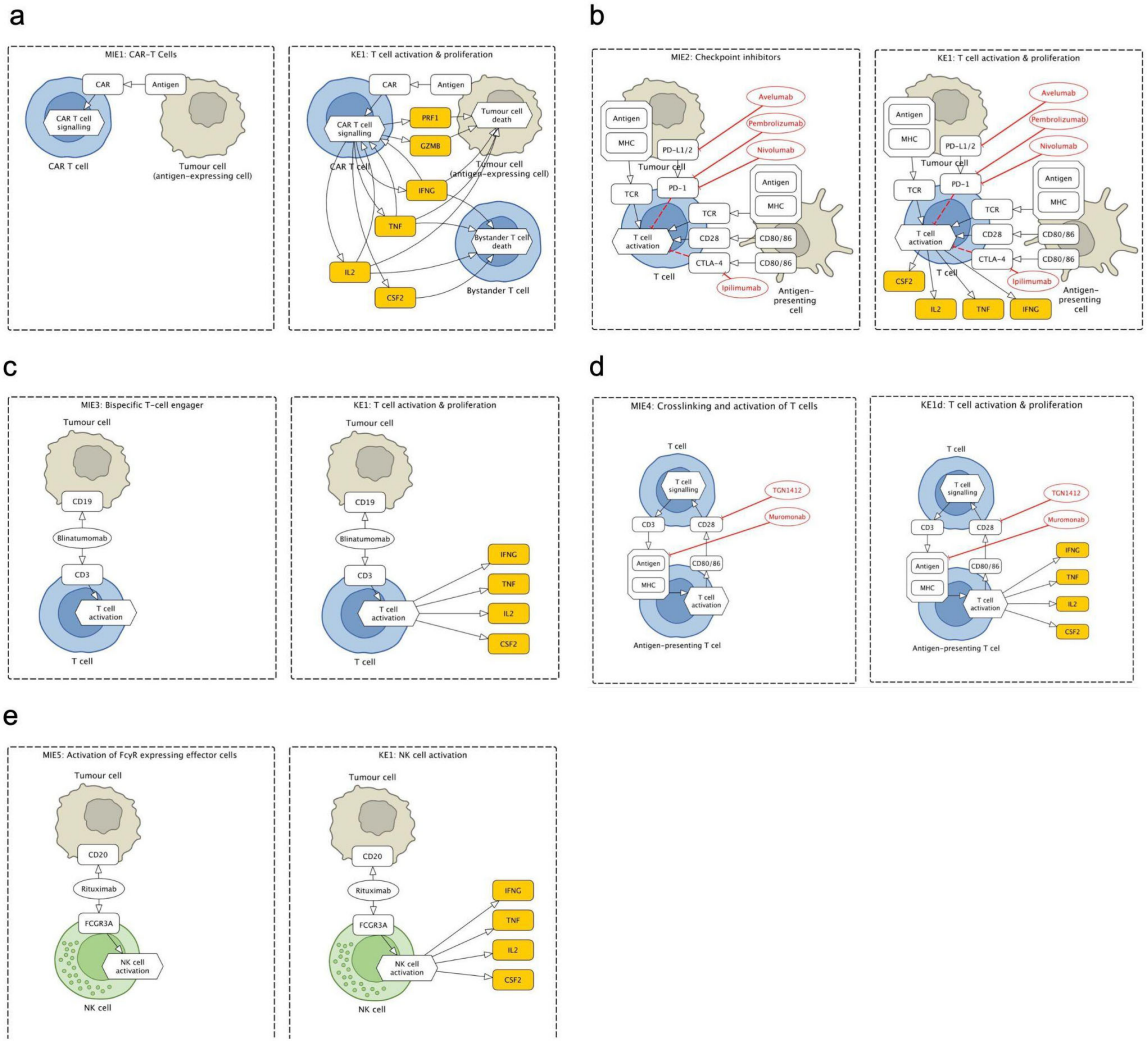
Fragments of the Cytokine Release Syndrome Map (CRS Map): molecular initiating events (MIEs) and key events (KEs) for five therapies that lead to cytokine release syndrome (CRS) as an adverse outcome (AO). **(a)** CAR T cells therapy: binding of CAR to antigen. **(b)** Checkpoint inhibitors (CPIs): binding of CPI to target T cell and formation of immune synapse. **(c)** T-cell engagers (TCEs): binding of TCE to target T cell and antigen expressing cells. **(d)** Crosslinking and activation of T cells: binding of T cell agonist mAb to T cell. **(e)** Activation of FcγR-expressing effector cells: binding of mAb to an antigen-expressing cell.

Together with the domain experts, we drafted the graphical components of the map (see Methods) and reconstructed an underlying network of molecular and cellular interactions for each KE separately. Then, we aligned KEs and connected them by matching the input and output molecules of subsequent KEs (**Figures 2**, **3**). During this network reconstruction, we identified and addressed knowledge gaps within and across KE mechanisms.

**Figure 4:**
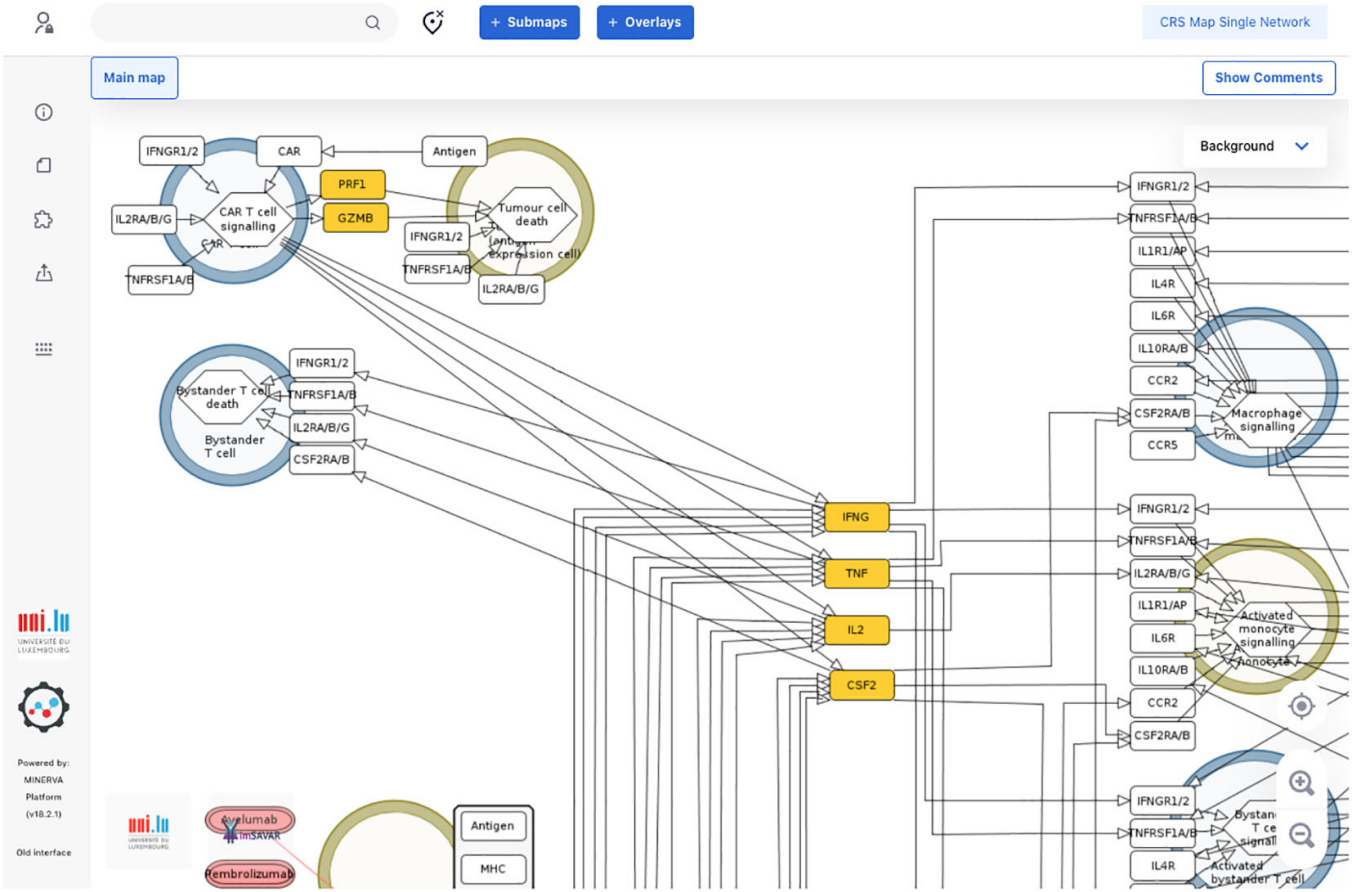
A fragment of a single-network version of the CRS Map in the MINERVA platform. This version is derived from and synchronised with the main CRS Map (**Figure 2**) and suitable for computational modelling and network analysis approaches. A single-network version for modelling is available online for browsing and exploration via the MINERVA platform at https://imsavar.elixir-luxembourg.org/minerva/?id=crs115. The map can be downloaded from MINERVA in various formats including SBML and CellDesigner formats (Submaps>Download).

The map was then encoded in a systems biology format, compliant with SBML and SBGN standards, enabling versioning, reproducibility and comparability. Finally, the map was published on the MINERVA Platform in its AOP version (https://imsavar.elixir-luxembourg.org/minerva/index.xhtml?id=CRSmap123) and single-network version (https://imsavar.elixir-luxembourg.org/minerva/?id=crs115). Both maps are publicly shared via the MINERVA-Net repository (https://minerva-net.lcsb.uni.lu/table.html) (43). **Figure 3** shows fragments of the discrete version and **Figure 4** shows a fragment of the single-network version.

### 2.3 Application of the CRS irAOP map

The CRS Map allows visual exploration of complex datasets because of its computer-readable format, stable identifiers and online access. To demonstrate the utility of the Map, we used two publicly available datasets (44, 45) to visualise differences in cytokine levels in CRS adult patients i) compared to healthy controls irrespective of the CRS grade, and ii) between high (4-5) and low (0-3) CRS grades. See Methods for details on dataset preparation and instructions on how to visualise them.

Teachey and coauthors (44) measured different soluble mediators in sera of healthy controls as well as in patients (children and adults) treated with the CTL019 CAR-T product (tisagenlecleucel) which was the first CAR-T product to be approved for market use by the U.S. FDA in 2017 and is sold under the name “Kymriah”. They observed that several peak values of cytokines, including IL-6, IFN-γ, sgp130 (natural inhibitor of soluble interleukin-6 receptor trans-signalling responses), and the soluble IL-6 receptor were associated with severe CRS.

**Figure 5:**
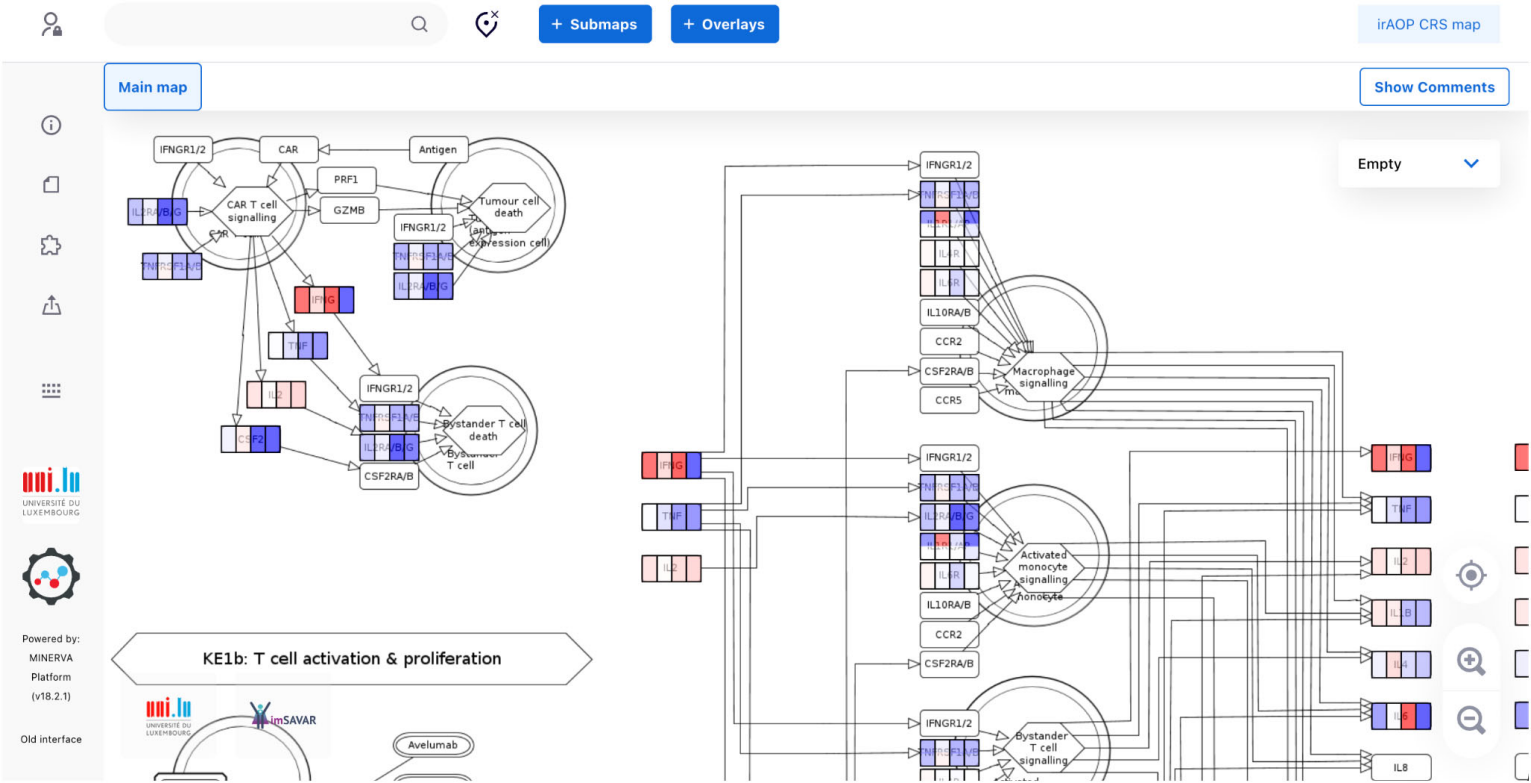
A fragment of the CRS map with the data from Teachey and coauthors visualised for cytokine expression profiles, as explained in the text. The full map with the four overlays is available for interactive exploration online, with a static image available in **Supplementary Figure S2**.

These results are represented in the CRS Map such that you can follow the levels of the different cytokines that are correlated with the respective CRS grade. Visual exploration is possible via the OVERLAYS tab in the map (https://imsavar.elixir-luxembourg.org/minerva/index.xhtml?id=CRSmap123), by selecting one or more of the prepared datasets visible in the left panel. Four overlays are available, for cytokine expression profiles of i) adults vs controls at baseline, ii) children with high vs low burden at baseline, iii) adults of high vs low grade CRS at day 1–3 peak values, and iv) adults of high vs low grade CRS at one month peak values (**Figure 5**, **Supplementary Figure S2**).

Further, we analysed time series data describing the expression of key CRS markers depending on grade (45). In this study, patients were treated with a defined 1:1 ratio of CD4:CD8 CD19 CAR-Ts (clinical trial: NCT01865617). Here, the authors observed that patients who developed high-grade CRS showed high peak values of e.g. IL-6, IFN-γ and MCP-1 early after CAR-T cell infusion (day 1-3) but also up to day 30 after CAR-T infusion compared to patients who only showed mild CRS symptoms. This product is now also approved for market use and is sold under the name “Breyanzi” (lisocabtagene maraleucel).

Here, the results of the study are also colour-coded in the CRS Map. Similarly, they are available in the OVERLAYS tab at https://imsavar.elixir-luxembourg.org/minerva/index.xhtml?id=CRSmap123, enabling the user to follow how the cytokines peak at the different time points and which cell types are involved from the CRS initiating event to the adverse outcome. Overall, eight datasets are available, four for non-severe and four for severe CRS. Time points were grouped in pairs to visualise high-level trends in the time series (static image is available in **Supplementary Figure S3**).

In practice, the CRS map can be a part of computational analysis of AOP omics readouts, in particular using application programming interface (API) offered by the MINERVA Platform (46). Such a workflow, for instance, can automatically process proteomic readouts of an AOP model, and upload them to the CRS map for visual inspection to identify interactions between key molecules, or important temporal changes in relevant KEs. Finally, AOP data and CRS map can be combined into a computational model, enabling simulation of specific outcomes under proposed perturbations or interventions (47).

## 3 Discussion/outlook

The CRS Map supports identifying strategies to minimise or monitor damaging effects of potential side effects of novel immunomodulatory therapies at an early stage, i.e. prior to first-in-human studies. To construct the CRS Map, we are the first to combine the AOP concept of systems toxicology (2, 48) with the disease maps approach of systems biology (13, 49). The AOPs describe the side effects of a treatment by outlining the chain of events that lead to an adverse outcome, relevant cell types, and various molecules that are measurable in *in vitro* or *in vivo* models, along with clinical biomarkers. Thus, an AOP integrates test systems that can mimic these key events. Information on various AOPs can be found in AOP-Wiki (http://aopwiki.org), the main platform for systematically organising, storing and sharing AOPs. Importantly, several AOPs can be combined to form an AOP network, however at the cost of increased complexity and challenges with harmonisation. To address this, we applied the disease maps paradigm to AOPs involved in CRS, introducing systems biology formalism, stable identifiers for involved cell types and molecules, diagrammatic representation, and a computational endpoint for the AOP-based CRS model.

While developing the CRS Map, we had to balance the advantages of the comparatively compact high-level human-readable representation and the related disadvantages of not providing more detailed information for relevant mechanisms including information about pathways inside the cells involved. For example, we can distinguish CD4+ and CD8+ T cells on the map but this would multiply similar interactions, limiting map readability. Also, specific mechanisms that involve PRF1 and GZMB in CAR T setting can be described in more detail but this process needs to be done consistently for all mechanisms, together with an improved design matching the increased complexity. A potential solution is adding additional layers of granularity similar to how, for example, the AsthmaMap resource is organised (https://asthma-map.github.io) (50). The navigation within such a multi-layered resource is described in the tutorials at https://asthma-map.github.io/tutorials. This multi-layered representation can be further developed in the future for the CRS model and text mining-based solutions can be explored for this purpose to ensure scalability of map updates (51).

Another direction for further development is the role of metabolites in CRS. The current CRS model does not explicitly include metabolic components. For CAR−T cell therapy studies, evidence shows that metabolites are linked to CRS onset, severity, and cytokine dynamics (52, 53). Omega‐3 fatty acids are suggested to be linked to CRS (54). Due to limited mechanistic clarity directly connecting metabolites to specific molecular initiating or key events to CRS, they were not incorporated in this iteration, though this direction is important to explore for future model refinements.

Immunotherapies are powerful approaches for the treatment of a broad range of pathological conditions. Nevertheless, they can be linked to a variety of adverse side effects, such as CRS, based on complex immunological mechanisms occurring in the patient upon therapeutic intervention. In the field of CRS induced by immunotherapies, many lessons have been learned. For instance, the disastrous outcome of the clinical trial phase I with TGN1412 induced a turning point regarding the design of first-in-human studies (55, 56). Also, due to intensive research on CAR T cell-induced CRS, up to now this adverse event can be managed quite well (42). Despite this progress, further steps remain to be made to reach the point where this adverse effect can be prevented or predicted. To this end, well-structured and reliable knowledge should be publicly available to fully exploit the lessons learned and e.g. translate them into improved nonclinical assessment, define follow-up research questions, or identify suitable predictive biomarkers. This becomes possible with the large amount of relevant literature and the current research focus shifting towards a generation of large genomic, transcriptomic, and proteomic datasets. Insights gained by novel data can be put into context by integration into the CRS Map (44–46, 50, 52, 53, 57).

During the first imSAVAR study-a-thon, working as a multi-disciplinary group of experts, we combined CRS-related irAOPs and set up a MINERVA-based disease map to organise the existing body of knowledge, harmonise it, and make available for data visualisation and computational analysis. As demonstrated here, it resulted in the generation of an easily accessible interactive knowledge base, which can be used for different purposes. In particular, we harmonised the AOPs for five CRS-inducing immunotherapies. The comprehensive representation of the complex network structures given by the irAOP in the disease map facilitates the identification of common key players, e.g. molecules or cell types, or shared “pathway hubs”. Thus, despite the differing MIEs and modes of action, it can be a valuable source for novel research strategies to uncover pathophysiological mechanisms representing the key event relationships (KERs). The CRS Map can also guide the development of nonclinical models for predictive safety assessment, which renders them as a basis for the improvement of safety-efficacy profiles of powerful immunotherapies. Moreover, the knowledge represented by the map can support uncovering mechanisms of other well-known causes of CRS, such as sepsis or viral infections with e.g. influenza or SARS-CoV-2, as they might share similar pathway hubs despite the different MIE (4, 58).

By clearly defining the individual cell types and soluble mediators involved in each key event, as approached here and in future work, the suitability of individual (immunotoxicological) assays to predict adverse outcomes can be assessed. For example, multiple assay formats have been developed and are used to predict the occurrence of CRS during the drug development process. In our harmonised AOPs, we can see that classical cytokine release assays that use whole blood or PBMCs (59) to measure cytokine levels after treatment with a drug will only be able to capture the biological processes in KE1 and KE2. However, introducing more complexity by e.g. incorporating endothelial cells in wet-lab models and measuring endpoints such as vascular leakage, would allow a model to capture later key events. For CRS, understanding at which key event the inflammatory response switches from a local physiological reaction to the pathological reaction would allow the development of improved *in vitro* models that would enable better adverse outcome prediction and potentially improved mitigation strategies.

## 4 Methods

### 4.1 Determining map coverage

To harmonise information across AOPs, domain experts built an AOP connectivity table (**Supplementary Table S1**) as a working file that reflected the information from the AOP network for the five treatment strategies as shown in **Figure 1** and **Table 1**. The supporting references were stored in a shared Zotero library.

The AOP connectivity table (**Supplementary Table S1**):

- identified molecules shown to be involved in the mechanisms of cytokine release syndrome via connecting to HGNC names and UniProt entries;
- determined source and target cell types for key molecules, ensuring connectivity in the CRS Map;
- identified receptors for each of the molecules where applicable;
- referenced supporting publications by providing PMIDs;
- highlighted the biological significance of each molecule in the context of cytokine release syndrome.

This table can be considered the main tool for successful communication between the expert team and the map development team, as well as the main environment for preparing a consistent and structured high-quality representation of relevant biological mechanisms.

When the initial version of the CRS Map was available, the diagram and the table were reviewed to identify inconsistencies and gaps. The changes were reflected in the summary table (**Supplementary Table S1**), in the Zotero library, and in the CRS Map.

### 4.2 Literature search

The CRS Map was built according to the community guidelines (14) and a literature review was conducted in PubMed to support interactions in **Supplementary Table S1**, to determine map coverage and to further extend the map. The search was done for papers published between 1980 and 2024, focusing on such topics as irAOPs, CRS and mechanisms specific for the MIEs. The type of literature search was in line with the scoping review strategy (14) to systematically identify key cell types, pathways and molecules involved in the MIEs, KEs and the adverse outcome. A variety of search strategies was used to ensure comprehensive coverage. The search process was iterative and adapted to different aspects of the topic. For example, general queries included terms such as “cytokine release syndrome” AND immunotherapy AND mechanisms or “immunomodulatory biotherapeutics” AND “toxicity”. For narrower, therapy-specific queries, examples include “CAR-T” AND mechanisms AND “cytokine release syndrome” and (“checkpoint inhibitors” OR CPIs) AND mechanisms AND “cytokine release syndrome”. These searches were refined through combinations of MeSH terms and free-text keywords, and the resulting literature was manually curated for relevance to the construction of the map and the underlying mechanistic representation.

For future iterations, text mining tools can be a valuable addition, particularly to expand coverage, identify overlooked interactions or prioritise areas for expert review.

### 4.3 Editors and data model

The freely available yEd Graph Editor (https://www.yworks.com/products/yed) was used for building the initial version by applying the SBGN palette (60). A CellDesigner version of the map was manually redrawn to allow easier compatibility for editing. While there was an option to use an automatic conversion to SBGN-ML via the ySBGN tool (61), manual redrawing was chosen to improve the layout and to revise the map against the connectivity table (**Supplementary Table S1**).

The data model follows the SBGN schema (62) for Activity Flow diagrams (63). To allow compatibility, the logic of the reduced notation of CellDesigner is used, and the rounded rectangle shape is used for proteins instead of the rectangle shape as in the standard Activity Flow language. For improved visual exploration, background images are used to show different cell types, which are not part of the SBGN notation.

To avoid showing in detail known intracellular signalling pathways, phenotype glyphs were used to represent submaps, for example, ‘Dendritic cell signalling’ for the events in dendritic cells. Phenotype glyphs were also used to show the final outcomes, for example, ‘Vascular leakage’.

### 4.4 Designing map structure

For the CRS Map, a workflow was introduced and tested, where the biological mechanisms are represented separately for each corresponding event of the AOP network. The mechanisms are initially shown not as a single interconnected network but as several subnetworks where each subnetwork corresponds to one entity of the AOP network. For comparison, see MIE1 and KE1a in the CRS AOP network (**Figure 1**) and the CRS Map (**Figures 2**, **3**). At the same time, a single-network version was also developed to allow modelling and network analysis approaches (**Figure 4**).

To structure these ‘snapshot’ subnetworks and connections between them:

1. the output molecules of one step become the input molecules of the next step;
2. the appropriate cell types are positioned in the middle of the ‘snapshot’ subnetwork diagrams with input molecules on the left side and output molecules on the right side.

This approach is based on the disease maps construction protocol (14) adapted for AOPs (64), and was implemented during a dedicated study–a-thon (see below).

### 4.5 Study-a-thon

Interdisciplinary work required for map design and curation was done during a dedicated study-a-thon, in a hackathon-style (65) hands-on workshop focused on research organised as a part of the IMI2/EU project imSAVAR (https://imsavar.eu). The study-a-thon was a four-day meeting during which domain experts systematically identified cell types and molecules implicated in CRS AOPs, which were then encoded with stable identifiers and literature references. Each day of the study-a-thon was split into domain-specific work and interdisciplinary exchanges allowing the AOP experts team and the computational biology team to establish identifier tables, create the integrated AOP network (**Figure 1**) and build the base of the connectivity table (**Supplementary Table S1**).

### 4.6 Publishing the map online

To make the diagram easily accessible online and to enable exploration and data visualisation of the resource, the CRS Map was uploaded to the MINERVA platform (66). The functionalities of the platform and its application examples are covered in previous publications (14, 43, 46, 66).

### 4.7 Resource sustainability and updates

The main development phase of this work was carried out during a period of dedicated support within the imSAVAR project. Such conceptual models are evolving dynamic resources, which should be kept accessible and updated. The map is hosted on the MINERVA platform, which is actively developed and used across multiple projects. The imSAVAR CRS map is hosted by ELIXIR Luxembourg, ensuring at least 10 years of sustained availability. The CRS map is shared the MINERVA Net registry (https://minerva-net.lcsb.uni.lu) (43) and published on the disease-maps.io website, ensuring visibility of the resource.

Updates are aligned with extensions of the imSAVAR project, or carried via partner or community efforts. The model is openly available under a Creative Commons Attribution 4.0 International License (CC BY 4.0), allowing other groups to reuse and further develop the resource.

### 4.8 Data analysis and visualisation

Data describing the expression of CRS-related cytokines were obtained from (44). Cytokine expression for adults compared to controls was obtained from Supplementary Table 7 from the article, while a comparison of peak cytokine levels between high (4-5) and low (0-3) CRS grades was obtained from Supplementary Table 9 and Supplementary Table 11, respectively (44). Only median values were used. CRS levels were normalised to [-1,1] range for visualisation purposes. We rounded the values to [-3,3] range and divided them by 3, to reduce the impact of the high expression values on the normalisation of the entire dataset.

Data describing time series expression of CRS-related markers were obtained from the publication by Hay and coauthors (45). Data from Supplementary Figure S5 was obtained by PlotDigitizer (https://plotdigitizer.com) for Non-Hodgkin lymphoma (NHL), for cytokines MCP-1, IFN-γ and IL-6. Time points were grouped in pairs (1 and 2, 3 and 4, 5 and 6, 7 and 8), and expression values were averaged. The last time point was omitted. Values were normalised by dividing by the max value.

The datasets can be explored together with the map contents interactively via the functionalities of the MINERVA Platform. For details on how to display data overlays and examine expression values for individual molecules, see the tutorial video at https://minerva.pages.uni.lu/doc/quickstart/, section “Backgrounds and general overlays”.

## 5 Conclusion

The novel systems immunotoxicology approach of integrating the AOP framework with the disease maps methodology offers a digital model - the CRS Map - for an improved understanding of immunological mechanisms underlying CRS at molecular and cellular levels. This model facilitates a more intuitive exploration for domain experts and opens possibilities for data analysis via the MINERVA platform. The value of the work is in a novel integrated systems immunology methodology in the research of adverse outcomes, and in offering an explorable online digital model of the CRS mechanisms. By systematically mapping out the adverse outcome mechanisms, this digital model can aid in understanding and accessibility of the knowledge by different domain experts and mitigate the risks associated with immunomodulatory biotherapies, improving patient safety and therapeutic efficacy. Additionally, it can be a tool to understand additive risk for combination therapies, as well as hypothesis generation for novel therapies with CRS observations. The online CRS Map resource allows applications for data visualisation and guiding intervention strategies.

## Data Availability

The datasets presented in this study can be found in online repositories. The names of the repository/repositories and accession number(s) can be found in the article/**Supplementary Material**.
