# Supplementary figures and images for: An explorable model of an adverse outcome pathway of cytokine release syndrome related to the administration of immunomodulatory biotherapeutics and cellular therapies
Source: Front Immunol. 2025 Aug 8;16:1601670. doi: 10.3389/fimmu.2025.1601670 (PMC12371706; doi:10.3389/fimmu.2025.1601670)

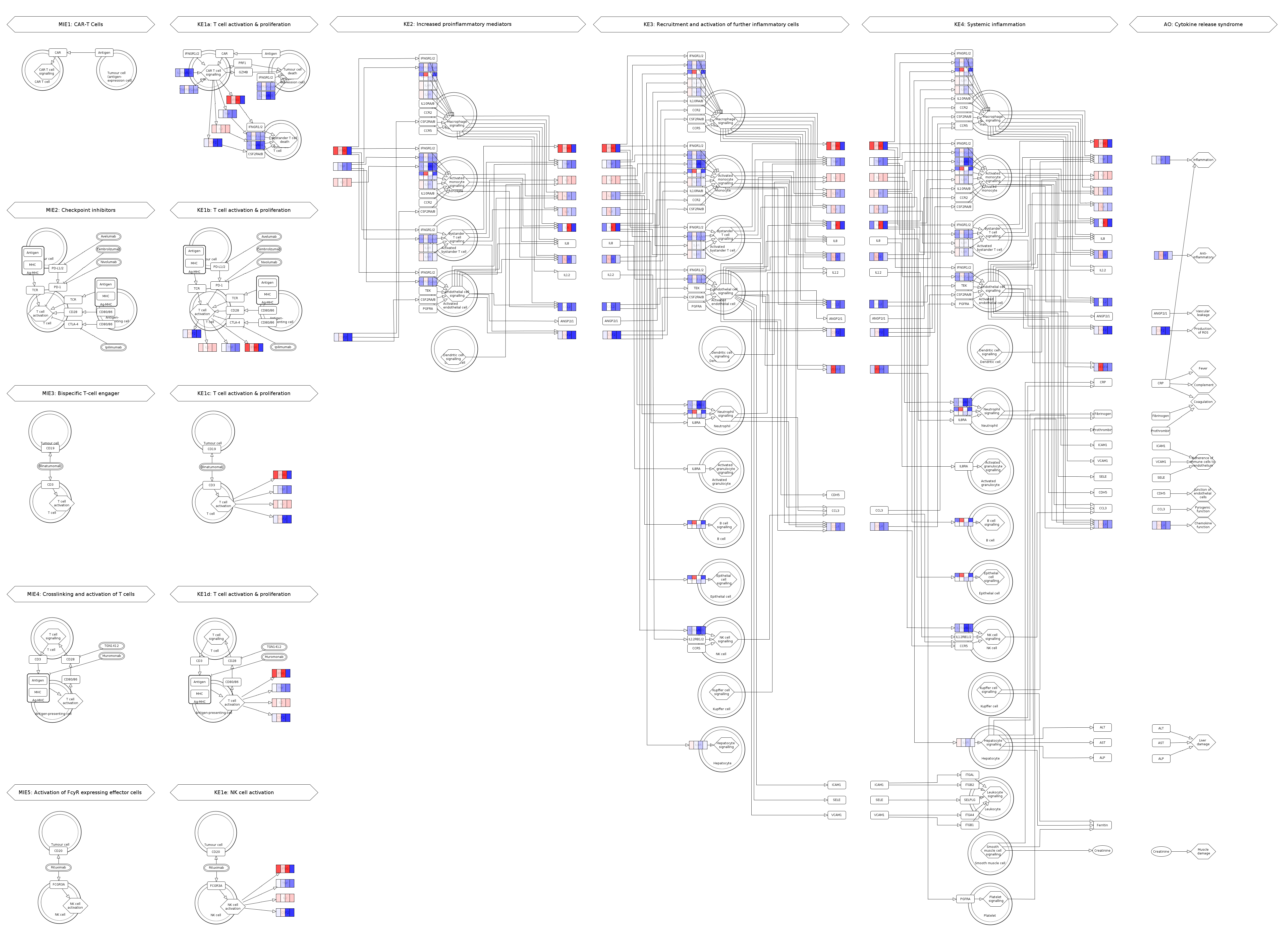

Supplement: Supplementary file 2 [file Supplementaryfile2.png]

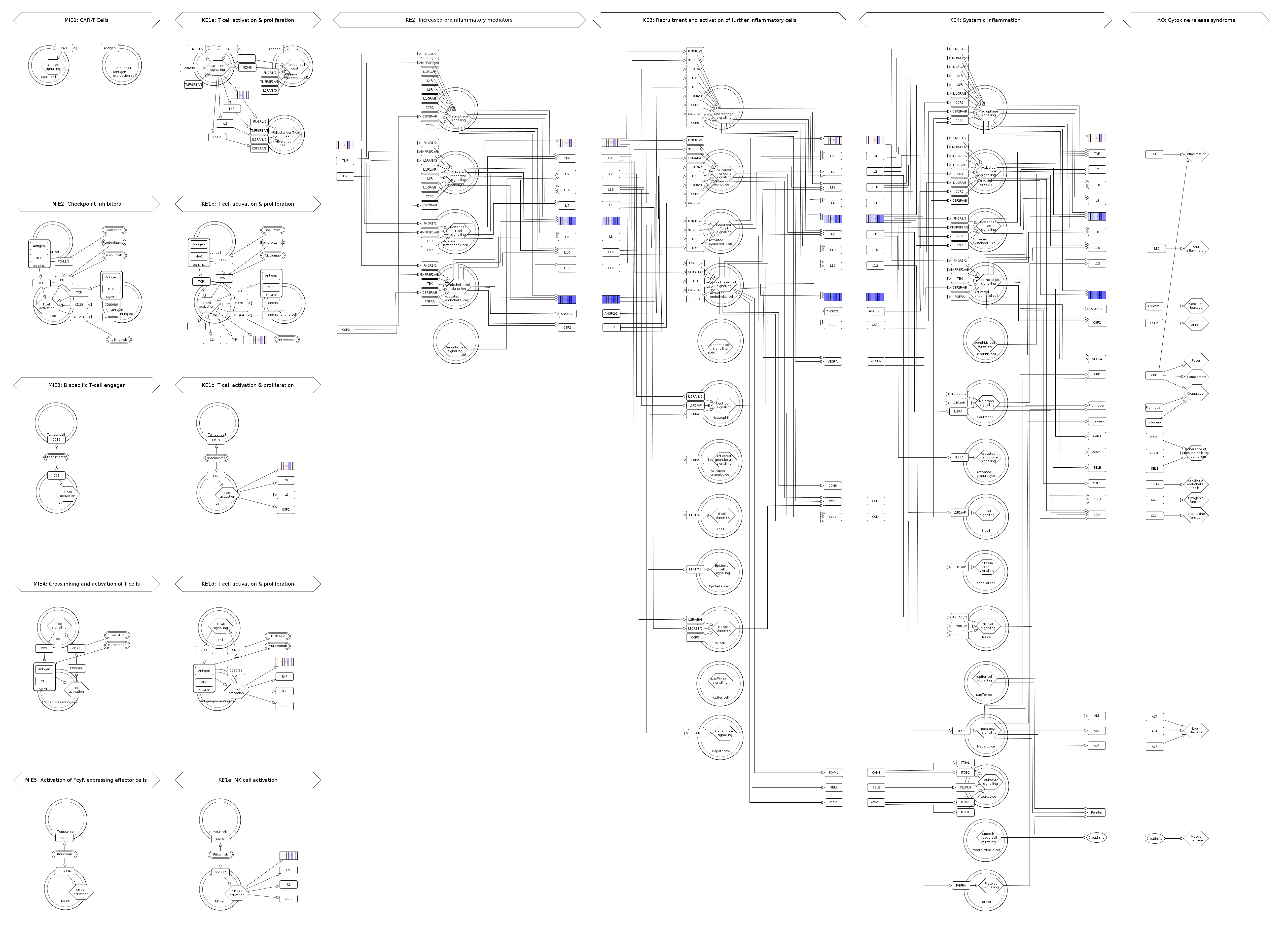

Supplement: Supplementary file 3 [file Supplementaryfile3.png]
